# Prevalence and Clindamycin Resistance Profile of *Staphylococcus aureus* and Associated Factors among Patients Attending the University of Gondar Comprehensive Specialized Hospital, Gondar, Northwest Ethiopia

**DOI:** 10.1155/2022/6503929

**Published:** 2022-06-14

**Authors:** Aklilu Ambachew, Teklay Gebrecherkos, Getnet Ayalew

**Affiliations:** ^1^Department of Medical Microbiology, School of Biomedical and Laboratory Sciences, College of Medicine and Health Sciences, University of Gondar, Gondar, Ethiopia; ^2^Department of Medical Microbiology and Immunology, College of Health Sciences, Mekelle University, Mekelle, Ethiopia

## Abstract

Clindamycin can serve as an alternative treatment for staphylococcal infections. Routine susceptibility tests may fail to determine inducible type clindamycin resistance and can be a source of failure in clinical therapeutics. Therefore, this study aimed to determine *Staphylococcus aureus* (*S. aureus*) prevalence, inducible clindamycin resistance pattern, and associated factors among patients attending the University of Gondar Comprehensive Specialized Hospital, Gondar, northwest Ethiopia. *Methods*. A cross-sectional study was conducted from January to April 2018. Clinical samples were inoculated on appropriate culture media. Standard bacteriological tests, including Gram stain, catalase, and coagulase tests, identified the presence of *S. aureus*. The antimicrobial susceptibility tests and the *D*-test were performed by using the Kirby–Bauer disk diffusion technique on the Mueller–Hinton agar. The *D*-test was performed using clindamycin (CLI) 2 ug and erythromycin (ERY) 15 ug disks located approximately 15 mm apart, and the cefoxitin susceptibility test was used to characterize methicillin-resistant *S. aureus* (MRSA). The association between *S. aureus* infection and different variables was assessed using bivariate and multivariate analysis. A *P* value <0.05 was considered statistically significant. *Result*. Of 388 study participants, the overall prevalence of *S. aureus* was 17% (66/388). Of these, the inducible type of clindamycin resistance was 25.8% (17/66) and 21.2% (14/66) were MRSA. All isolates were susceptible to chloramphenicol and resistant to tetracycline. A family size of 4–6 (AOR = 2.627, 95% CI (1.030–6.702)) and >7 (AOR = 3.892, 95% CI (1.169–12.959)), inpatient study participants (AOR = 3.198, 95% CI (1.197–8.070)), illness in the previous 4 weeks (AOR = 2.116, 95% CI (1.080–4.145)), and a history of chronic disease (AOR = 0.265, 95% CI (0.094–0.750)) were likely to have *S. aureus* infection. *Conclusion*. This study shows a considerable high magnitude of MRSA and inducible clindamycin resistance *S. aureus* isolates. To rule out clindamycin susceptibility testing, the *D*-test should be routinely performed.

## 1. Introduction

*Staphylococcus aureus* (*S. aureus*) is found in about 30% of healthy people as flora in various body parts, including oral cavity, nasal passages, throat, intestines, skin, and mucous membranes and can be carried by the host for a long period of time without causing clinical consequences [1–5]. However, *S. aureus* has the ability to cause a broad spectrum of serious infections, starting from a comparatively mild involvement of the skin and soft tissue, to life-threatening systemic illnesses such as pneumonia, meningitis, bloodstream infections, and endocarditis, as well as toxin-mediated syndromes such as toxic shock, scalded skin syndrome, and food poisoning, are associated with significant morbidity and mortality [4,5]. It is also a predominant cause for nosocomial-acquired infections such as intravenous catheter-associated infections, ventilator-associated pneumonia, postsurgical wound infections, invasive infections in neutropenic patients, and patients undergoing solid organ or hematopoietic cell transplantations [6,7].

Macrolide-lincosamide-streptogramin B (MLSB), a family of antibiotics, such as erythromycin (ERY), clindamycin (CLN), and streptogramin B, serves as an alternative for the treatment of staphylococcal infections. Owing to its excellent pharmacokinetic properties and ability to treat methicillin-resistant *S. aureus* (MRSA), CLN is the preferred agent [8,9]. However, the widespread use of the MLSB family of antibiotics has led to an increase in the number of staphylococcal strains acquiring resistance to these antibiotics [10]. The MLSB resistance occurs by different mechanisms, including an active efflux mechanism that is encoded by the MSRA gene (macrolide streptogramin resistance A), conferring the MS phenotype. These strains appear CLN sensitive and ERY resistant *in vitro* and do not become CLN-resistant during therapy [11,12]. Methylation of the ribosomal target site is mediated by the erm gene (erythromycin ribosome methylase) encoding an rRNA methylase enzyme that inhibits protein synthesis by binding to the 50S ribosomal subunit. This mechanism can be either constitutive macrolide-lincosamide-streptogramin B (cMLSB), where this enzyme is always produced or inducible macrolide-lincosamide-streptogramin B (iMLSB), where an inducing agent is required for its production [13,14]. Erythromycin is a strong inducer of methylase synthesis, while lincosamides such as clindamycin are comparatively weak inducers [15].

Antimicrobial susceptibility testing of *S. aureus* isolates with a constitutive phenotype reveals resistance to both ERY and CLN. On the other hand, those with an inducible phenotype are resistant to ERY but appear susceptible to CLN if not placed adjacent to each other *in vitro*. Therefore, treating patients with CLN in such circumstances leads to the emergence of constitutive erm mutants, and this causes clinical and therapeutic failure [16]. iMLSB is not recognized by most standard antimicrobial susceptibility testing methods, including the standard broth-based method, the standard agar dilution method, and other automated systems [17]. The iMLSB phenotypes are only detected by the disk diffusion technique with placement of a strong inducer ERY and CLN adjacently with a 15 mm diameter on the Muller–Hinton agar (MHA) and after overnight incubation shows a “D” shaped zone of inhibition which indicates its resistance *in vitro* induction test (“D” test) [18–20]. Some of the identified possible predisposing factors for acquisition of macrolide-lincosamide-streptogramin B and methicillin antimicrobial resistance in *S. aureus* are indiscriminate and excessive antibiotic usage, prolonged hospital stays, presence of chronic diseases, lack of awareness, lack of personal hygiene, history of catheterization, history of hospitalization, and proximity to an already infected or colonized patient [1,21,22].

According to a meta-analysis study, the prevalence of MRSA in Ethiopia was 32.5% [23] and the prevalence of *S. aureus* resistance to different antimicrobial agents in Ethiopia showed *S. aureus* resistance to vancomycin was 11%, *S. aureus* resistance to ciprofloxacin was 19%, *S. aureus* resistance to erythromycin was 41%, and *S. aureus* resistance to Clindamycin was 24% [24]. Scant information is available on the clindamycin resistance profile of *S. aureus* in Ethiopia. Thus, the present study aimed to investigate the prevalence of *S. aureus* and clindamycin resistance profiles, as well as associated factors, in patients attending the University of Gondar Comprehensive Specialized Hospital, Gondar, northwest Ethiopia.

## 2. Methods

### 2.1. Study Design Area and Period

An institution-based cross-sectional study was conducted at the University of Gondar Comprehensive Specialized Hospital, Gondar town, northwest Ethiopia, from January to April 2018. Gondar town is located in the northwest part of Ethiopia and is 748 km from Addis Ababa. The University of Gondar Comprehensive Specialized Hospital is a referral hospital with more than 500 beds and serves a population of more than 5 million. It is one of the biggest tertiary-level referral and teaching hospitals in the region. The hospital consists of different units, including an intensive care unit (ICU) with 18 beds, 13 wards with 510 beds, outpatient departments, and the diagnostic laboratory. The diagnostic laboratory is divided into different sections. Culture and antimicrobial susceptibility testing are among the hospital services in the medical microbiology section [25].

### 2.2. Population, Sample Size, and Sampling Technique

All outpatients and inpatients who were requested by physicians for the routine microbiological culture and the antimicrobial susceptibility test were included in the study. Patients who had taken antibiotics in the past four weeks were excluded from the study. The sample size was determined by using a single population proportion formula: *n*=(*z*2*p*(1 −*P*)/*d*2). Due to lack of previous studies in the study area, the 50% prevalence (*P*=0.5) is used to calculate the sample size with a 95% confidence interval (*z* = 1.96) and a 5% marginal error (*d* = 0.05). Therefore, the sample size for this study was 384. A systematic random sampling was used to select the study participants. A systematic random sampling was used to select the study participants. A review of two years of laboratory records prior to the study period showed that on average, 14 clinical samples (i.e., urine, blood, swabs, and other body fluids) had been recruited for the culture and antimicrobial susceptibility test per day. Accordingly, the total number of participants who would be included in this study in a 3-month period was calculated at 1,260. Therefore, the sampling interval (*K*^th^ interval) was calculated as *N*/*n*; where: *N* = the total estimated number of samples in three months and *n* = the required sample size. Hence, the sampling interval was drawn at 3. The first patient was selected by the lottery method and the rest of the study participants were selected every 3 patients.

### 2.3. Data Collection, Sample Collection, and Processing

After obtaining written informed consent, data on sociodemographic factors (age, sex, educational status, family size, and residence) and associated factors for acquisitions of *S. aureus* infection (healthcare worker family, history of hospitalization, and history of previous antibiotic use) were collected from each study participant and/or their parents/lawful guardians using a pretested structured questionnaire. All clinical specimens (blood, wound discharge, body fluids, urine, ear swabs, and eye swabs) were collected from various sites of infection and transported to the Medical Microbiology Laboratory Section of the University of Gondar.

### 2.4. Culture and Identification of *Staphylococcus aureus*

On arrival, all types of clinical samples were inoculated into mannitol salt agar (MSA) [23] (Liofilchem Ltd., Italy) and blood agar plates (BAP) (Oxoid Ltd., England) with sterilized wire loops to obtain discrete colonies [26]. The inoculated agar plates were incubated at 35°C for 24–48 hours under aerobic conditions. After overnight incubation, the inoculated media were examined for golden-yellow colonies on the MSA and *β* hemolytic, large colonies on the BAP that indicate the isolate could be *S. aureus*, which was subsequently identified by performing the Gram staining, catalase test, and coagulase test. Isolates that had shown Gram-positive cocci in clusters, catalase, coagulase, and mannitol fermentation positive were identified as *S. aureus* [26].

### 2.5. Antibiotic Susceptibility Testing

Antimicrobial susceptibility tests of isolates were performed by using the Kirby–Bauer disk diffusion technique on the Muller–Hinton Agar (MHA) (Liofilchem Ltd., Italy) [26] according to the Clinical and Laboratory Standards Institute (CLSI) [27]. The antimicrobial agents used include, cefoxitin FOX (30 *μ*g), clindamycin CLN (2 *μ*g), erythromycin ERY (15 *μ*g), chloramphenicol CAF (30 *μ*g), ciprofloxacin CIP (5 *μ*g), amoxicillin/clavulanate AMC (20/10 *µ*g), cloxacillin COX (5 *μ*g), tetracycline TTC (30 *μ*g), gentamicin CN (10 *μ*g), penicillin PEN (10units), trimethoprim-sulphamethoxazole SXT (25 *μ*g), doxycycline DOX (30 *μ*g), rifampin RIF (5 *μ*g), and nitrofurantoin NIT (300 *μ*g). The cefoxitin disk was used to characterize MRSA isolates. A zone of inhibition was measured in millimeters using a ruler around each antimicrobial disc. The results were reported as sensitive, resistant, or intermediate according to the CLSI recommendation.

### 2.6. *D*-Test Procedure

This test has been performed to detect the presence of inducible clindamycin resistance among ERY-resistant *S. aureus* isolates from patients. A bacterial inoculum suspension was prepared, and its turbidity was checked for its equivalence to 0.5 McFarland's standard and then was inoculated onto the Muller–Hinton agar plate (MHA). The disc of CLN 2 *μ*g (Hi Media, Mumbai) and ERY 15 *μ*g (Hi Media, Mumbai) was placed at a distance of 15–26 mm edge to edge as per CLSI guidelines, and the inoculated MHA plates were analyzed after 18–24 hours of incubation at 35°C [27].

#### 2.6.1. Interpretation of the *D*-Test

*Staphylococcus aureus* isolates showing circular zones of inhibition with a diameter of ≤13 mm for ERY and ≥21 mm for CLN without a D-shaped zone along ERY were interpreted as negative for inducible resistance (*D*-test negative). *S. aureus* isolates with an equivalent inhibitory diameter as aforementioned, with a D-shaped zone around CLN, were interpreted as positive for inducible resistance (*D*-test positive). Four types of phenotypes were observed, which are listed as follows:

1. Inducible clindamycin resistance (iMLSB) phenotype: in this phenotype, *S. aureus* isolates show a D-shaped zone of inhibition around the CLN disk while being resistant to ERY
2. Constitutive clindamycin resistance (cMLSB) phenotype: in this phenotype, *S. aureus* isolates were resistant to both drugs ERT and CLN
3. Macrolide streptogramin (MS) phenotype: *S. aureus* isolates exhibited resistance to ERY and were sensitive to CLN
4. Sensitive (S) phenotype: isolates of *S. aureus* were sensitive to ERY and CLN

### 2.7. Quality Control

The reliability of the study findings was certain by employing quality control (QC) measures throughout the entire process of the laboratory work. All materials, equipment, and procedures were adequately controlled. Preanalytical, analytical, and postanalytical stages of quality assurance were followed. The sterility of culture media was insured by incubating 5% of each batch of the prepared media at 37°C for 24 hours. Performances of all prepared media were also checked by visual inspection and inoculating standard American Type Culture Collection (ATCC) bacterial reference strains of *S. aureus* (ATCC 25923), *S. aureus* ATCC® BAA-976™ (D-zone test negative), and *S. aureus* ATCC® BAA-977™ (D-zone test positive). The *S. aureus* (ATCC 25923) and *E. coli* (ATCC 25922) reference strains were also used to check the quality of the antimicrobial disks [28]. A double data entry was used to maintain data entry quality.

### 2.8. Data Analysis

The data were entered, cleaned, and analysed using SPSS version 20 computer software. The associations between dependent and independent variables were analysed by logistic regression. The adjusted odds ratio (AOD) and the 95% confidence interval (CI) were used to measure the strength of an association. A *p* value of ≤0.05 was considered statistically significant. Finally, the results are presented in tables, figures, and words.

### 2.9. Ethical Consideration

Ethical approval was obtained from the Ethical Review Committee of the School of Biomedical and Laboratory Sciences, College of Medicine and Health Sciences, University of Gondar, Ethiopia. Similarly, the study was conducted in accordance with the ethical principles of the Declaration of Helsinki on human subjects. All the study participants were informed concerning the study verbally, and a written informed consent was obtained from each participant. All information was treated as strictly confidential and used for this study only. Positive results were communicated to healthcare providers.

## 3. Results

### 3.1. Sociodemographic Characteristics of the Study Participants

In the present study, a total of 388 study participants were included and 51.3% (199/388) of whom were male. The mean age of the study participants was 19.76 (±21.0) years. The majority, 30.9% (120/388), of participants were aged less than one year. Most of the study participants, 65.5% (254/388), were single and lived in the family number of 4 to 6 (62.4% (242/388)). Almost half, 46.4% (180/388), of the participants were illiterate, whereas 7% (27/388) of the participants had a university degree. Around 58.2% (226/388) of the study participants were unemployed, but 14.7% (57/388) of the study participants were government employees. The majority, 60.8% (236/388), of participants lived in urban areas, as shown in (Table 1).

Seventy-five percent (291) of the study participants were inpatients, and the rest 25% (97) of patients were outpatients. One hundred twenty-two (31.4%) of patients were from the pediatric ward. Half of the patients, 49.7% (193/388), have had an illness during the past 4 weeks. More than 60% of the patients (257/388) implanted medical devices. Only a few of the participants were 9.0% (35/388) admitted to the hospital in the same year. Twenty-two percent of participants had a history of chronic disease. Few participants have experience of using drugs without a prescription, and only 10.3% (40/388) of the patients have family members working in healthcare (Table 1).

**Figure 1:**
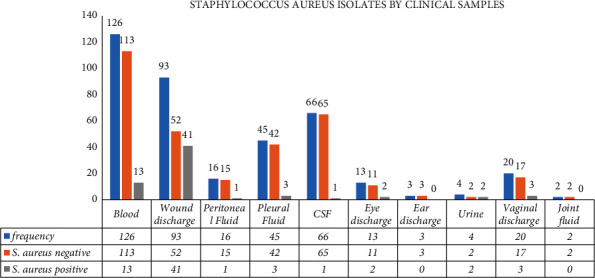
Frequency of *Staphylococcus aureus* isolates by clinical samples among patients attending University of Gondar Comprehensive Specialized Hospital, Ethiopia, 2018. CSF: cerebrospinal fluid.

Blood was the most common specimen type which is 32.5% (126), followed by wound discharge 23.9% (93), and CSF 17% (66). The urine specimen, ear discharge, and joint fluid were the less common type of specimens 1.0% (4), 0.8% (3), and 0.5% (2) that were processed, respectively (Figure 1).

### 3.2. Prevalence of *Staphylococcus aureus* among Patients Attending University of Gondar Comprehensive Specialized Hospital, Gondar, Northwest Ethiopia

From a total of 388 samples, 66 *S. aureus* isolates were isolated which makes the overall prevalence 17% (66/388). The highest proportion of *S. aureus* positivity was observed among females (53% (35/388)), aged between 11 and 20 years (27.3% (18/388)) and participants who were single (72.7% (48/388)). At the same time, 71.2% (47/388) of the participants who had a family size of 4 to 6. 33.3% (22/388) were illiterate, and 63.6% (42/388) who were unemployed had the highest prevalence of *S. aureus*. Moreover, the majority of *S. aureus* isolates were recovered from wound discharge specimens, and participants from inpatients, 62% (41/66) and 80.3% [29], respectively, especially patients from the pediatric ward were 39.4% [25] (Table 1 and Figure 1).

### 3.3. Prevalence of Macrolide-Lincosamide-Streptogramin B and Methicillin Resistance *S. aureus*

**Figure 2:**
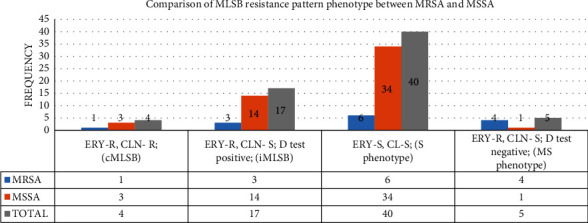
Comparison of the MLSB resistance phenotype between MRSA and MSSA among patients attending University of Gondar Comprehensive Specialized Hospital, Ethiopia, 2018.

From a total of 66 *S. aureus* isolates, 21.2% (14/66) were MRSA and the rest 78.8% (52/66) were MSSA. Inducible and constitutive types of clindamycin resistance were shown by 25.8% [16] and 6.1% [4] of *S. aureus* isolates, respectively. Among 14 MRSA isolates, 21.4% were the iMLSB type, only 7.1% [1] were cMLSB, 42.9% [30] were the S phenotype, and 28.6% [4] were the MS phenotype (Figure 2).

### 3.4. Antimicrobial Susceptibility Pattern of *S. aureus* Isolates

Regarding the drug susceptibility pattern, all S. *aureus* isolates showed a high rate of resistance to tetracycline 100% (66) and almost all 95.5% (63) to penicillin. Likewise, the majority of the S. *aureus* isolates showed a very high susceptibility to chloramphenicol 100% (66), nitrofurantoin 98.5% (65), gentamicin 92.4% (61), ciprofloxacin 92.3% (62), and rifampin 90.9% (60) (Table 2).

### 3.5. Associated Factors with *Staphylococcus aureus* Infection

In multivariate analysis, variables that were significant at the bivariate analysis at *p* value of 0.2 were included for the multivariate analysis. Accordingly those patients who had a family size of 4–6 (AOR: 2.627, 95% CI 1.030, 6.702) (*p*=0.043) and family size >7 (AOR: 3.892, 95% CI 1.169, 12.959) (*p*=0.027) were likely to have S. *aureus* infection than patients who had a family size of 1–3, and those inpatient study participants (AOR: 3.198, 95% CI 1.197, 8.070) (*p*=0.020) were likely to have S. aureus infection than outpatients, those patients who had illness in the last 4 weeks (AOR: 2.116, 95% CI 1.080, 4.145 (*p*=0.029) were likely to have S. *aureus* infection than those who had no illness in the last 4 weeks, and those patients who had a history of chronic disease (AOR: 0.265, 95% CI 0.094, 0.750) (*p*=0.012) were likely to have S. *aureus* infection than those who had no history of chronic disease (Table 3).

## 4. Discussion

*Staphylococcus aureus* is a significant human pathogen that can cause nosocomial and community-acquired infections. Global, national, and local studies have shown that *S. aureus* is among the primary bacterial species showing a high rate of multidrug resistance patterns because of its intrinsic ability to develop resistance to many antimicrobials [31].

The overall prevalence of *S. aureus* in this study was 17% (66), which was less than studies reported in Saudi Arabia at 40% and in South Africa at 25% [32,33] which was also lower than previous studies conducted here in Gondar, such as 23.4%, 23.9%, and 29.8% [34–36]. However, it was higher than the study reported by Tiruneh et al. and Gizachew *et al.* as 9.2% and 7.2% [37,38], respectively. The reason for this might be the sample size difference because our study sample size was small when compared with others, and the sample type used was known. The detection rate of *S. aureus* across various samples is considerably different.

The frequency of MRSA was 21.2% (14/66) in our study. This was in line with the study from Brazil, 20.7% [39] and 21.4% in Libya [40], but it is slightly higher than in Tanzania (16.3%) [41] and 17.6% in Gondar, Ethiopia [36]. In comparison with some other studies, the proportion of MRSA in this study was much lower than reported in Iran (44.4%), India (53.5%), and Nepal (53.6%) [19,42,43]. Such a variation might be due to differences in sample types, availability of drugs in the study areas, study participants involved, infection prevention practice, and unreasonable drug prescription.

In this study, 78.78% [44] of *S. aureus* isolates were MSSA, which is equivalent to the study result that was conducted in Brazil, 79.3% [39]. The proportion of MSSA was much higher than some of the studies conducted in Senegal (44.58%), India (35.3%) and (46.6%), Turkey (48.5%), and Iran (56%) [19,42,43,45,46]. The reason for such a difference in the distribution of MSSA in this study and other studies might be due to the variation in study participants, infection prevention practice, and irrational drug prescription culture.

The overall magnitude of inducible clindamycin resistance among *S. aureus* isolates of the study was 25.7% (17/66). The majority of inducible clindamycin resistance was found among MSSA at 26.9% (14/52). The frequency of inducible clindamycin resistance among MRSA isolates was 21.4% (3/14), and it is low when compared with MSSA. This finding was in concordance with a study conducted in Nepal, which is 21.1% [43]. This is higher when compared to other studies conducted in Libya (6.3%), in Iran (9.3%) and 10.4%, in Brazil (10.3%), and 15.4% in India [19,38,39,45,47]. However, the clindamycin resistance frequency (17.6%) among MRSA was incomparable with other studies conducted in Tanzania (61%), Nepal (27.9%), and India (36%) [8,42,46]. The higher proportion of inducible clindamycin resistance among MRSA might be due to its intrinsic ability, associated with the staphylococcal chromosomal cassette gene, and indiscriminate antibiotic usage.

The highest frequency of inducible clindamycin resistance was observed among 58.5% of male participants, 47.1% of aged 11–20, 82.4% who were single, 64.7% who had 4–6 family size, 30% who were primary school students, 70.6% who were unemployed, and 70.6% urban residents. Furthermore, there was a higher frequency of inducible clindamycin resistance observed in patients who had illness within the past four weeks (70.6%), history of surgical procedures (94.1%), wound infection (64.7%), inpatient admitted to hospital (76.5%), and history of chronic diseases (88.2%).

The cMLSB type of resistance in MRSA and MSSA was 7.1% (1/14) and 5.8% (3/52), respectively. The frequency of cMLSB in MRSA was corresponding to 8.9% in Libya [40], and it was insignificant when compared with 54.4% in Nepal, 69% in Brazil, and 77.6% in Iran [8,19,38]. This variation could be due to the number of *S. aureus* clinical isolates, in which in our study the number of *S. aureus* clinical isolates was 66, whereas it was higher in Nepal, Brazil, and Iran. The minimum number of *S. aureus* clinical isolates was 140.

In this study, the majority of MRSA isolates showed greater than 50% resistance to tetracycline, amoxicillin/clavulanate, penicillin G, erythromycin, doxycycline, and trimethoprim-sulphamethoxazole. However, more than 80% of the MRSA isolates in the study were susceptible to ciprofloxacin, nitrofurantoin, clindamycin, and chloramphenicol. This susceptibility result of ciprofloxacin was 78.6% higher than the studies conducted in India (52.2%) and Iran (64%) [16,48].

Interestingly, 92.9% of MRSA isolates were susceptible to clindamycin. It is a bit higher than the result in Gondar, at 67.6% [36]. Despite this, the use of clindamycin without *D*-tests may lead to therapeutic failure or may progress to drug resistance. In the study, among the iMLSB isolates, tetracycline, penicillin G, and doxycycline were resistant by 100%, 94.1%, and 88.2%, respectively. The iMLSB isolates were susceptible to trimethoprim-sulphamethoxazole by 64.7%, cloxacillin by 82.4%, ciprofloxacin, rifampin, and gentamicin by 94%, and nitrofurantoin and chloramphenicol by 100%.

In this study, the magnitude of multidrug-resistant *S. aureus* was 69%, which had been shown to be resistant to three or more types of antibiotics from different classes of antibiotics. All of the MRSA isolates in this study were multidrug resistant. The proportion of multidrug resistance among MRSA in this study was much higher than in some studies from different areas: 47% in Saudi Arabia, 65% up to 78% in Nepal, 84.8% in Addis Ababa, and 79.6% in Gondar [36,49–51]. And it was slightly higher than in Bahir Dar, at 96.8% [52]. This alarmingly high proportion of multidrug resistance among MRSA would call on hospital administration and health policymakers to pay attention to infection prevention and control. Furthermore, the need for strengthening the antimicrobial resistant stewardship program is required. The most common reason for the development of multidrug resistance is the irrational use of antibiotics without drug susceptibility testing and nonadherence to prescribed drugs by patients. In Ethiopia, there are some indications of irrational use of antibiotics by the community, patients, and healthcare providers [53].

In this study, *S. aureus* infection was significantly associated with patients' family size, currently admitted (inpatient) patients, patients who have had an illness in the last 4 weeks, and patients having chronic disease as compared to their counterparts. Due to the fact that overcrowding is favorable for the transmission of microbial infections, admission to a hospital may expose patients to nosocomial infections, including *S. aureus* infection. In addition, previous illnesses and chronic illnesses also have their own contribution to the transmission of *S. aureus* and the development of infection due to the lowered immunity in chronic disease patients [29,32,44,54].

## 5. Conclusion and Recommendation

This study showed a significant prevalence of *S. aureus,* and a considerable amount of MRSA was observed among *S. aureus* isolates. This study also found a high rate of the inducible clindamycin type of resistance among *S. aureus* isolates, and its frequency was more common among MSSA than MRSA. The antimicrobial susceptibility testing results showed that both methicillin-resistant *S. aureus* and inducible clindamycin-resistant *S. aureus* isolates were highly susceptible to ciprofloxacin, rifampin, chloramphenicol, nitrofurantoin, and gentamicin. Patients' family size, currently admitted (inpatient), illness in the last 4 weeks, and chronic disease were significantly associated with *S. aureus* infection. Due to resource limitations, this study was performed on phenotypic drug resistance patterns. The genotypic determination of drug resistance was left, and the study identification of *S. aureus* with the phenotypic method only is a limitation of the current study.

## Figures and Tables

**Table 1:** Sociodemographic characteristics and associated factors of the study participants with respect to the *Staphylococcus aureus* prevalence attending University of Gondar Comprehensive Specialized Hospital, Ethiopia, 2018.

Variables	Frequency *n* (%)	*Staphylococcus aureus*	
		Negative *n* (%)	Positive *n* (%)
*Sex*			
Male	199 (51.3)	168(52.2)	31(47.0)
Female	189 (48.7)	154(47.8)	35(53.0)

*Age (years)*			
<1	120 (30.9)	107(33.2)	13(19.7)
1–10	54 (13.9)	43(13.4)	11(16.7)
11–20	55 (14.2)	37(11.5)	18(27.3)
21–30	58 (14.9)	45(14.0)	13(19.7)
31–50	65 (16.8)	57(17.7)	8(12.1)
>50	36 (9.3)	33(10.2)	3(4.5)

*Marital status*			
Single	254(65.5)	206(64.0)	48(72.7)
Married	112(28.9)	97(30.1)	15(22.7)
Divorced	9(2.2)	7(3.0)	2(2.3)
Widow	13(3.4)	12(3.7)	1(1.5)

*Family size*			
1–3	82(21.1)	75(23.3)	7(10.6)
4–6	242(62.4)	195(60.6)	47(71.2)
>7	64(16.5)	52(16.1)	12(18.2)

*Educational level*			
Illiterate	180(46.4)	158(49.1)	22(33.3)
Primary	58(14.9)	43(13.4)	15(22.7)
Secondary	74(19.1)	58(18.0)	16(24.2)
College	49(12.6)	38(11.8)	11(16.7)
University	27(7.0)	25(7.8)	2(3.0)

*Occupation*			
Government	57(14.7)	48(14.9)	9(13.9)
Merchant	19(4.9)	15(4.7)	4(6.1)
Housewife	26(6.7)	23(7.1)	3(4.5)
Labour	26(6.7)	22(6.8)	4(6.1)
Farmer	34(8.8)	30(9.3)	4(6.1)
Unemployed	226(58.2)	184(57.1)	42(63.6)

*Residence*			
Urban	236(60.8)	188(58.4)	48(72.7)
Rural	152(39.2)	134(41.6)	18(27.3)

*Type of patient*			
Inpatient	291(75.0)	238(81.8)	53(18.2)
Outpatient	97(25.0)	84(86.6)	13(13.4)

*Illness in the last 4 weeks?*			
Yes	193(49.7)	149(77.2)	44(22.8)
No	195(50.3)	173(88.7)	22(11.3)

*Implanted medical device*			
Yes	257(66.2)	221(86.0)	36(14.0)
No	131(33.8)	101(77.1)	30(22.9)

*History of surgical procedure*			
Yes	28(7.2)	22(78.6)	6(21.4)
No	360(92.8)	300(83.3)	60(16.7)

*History of hospital admission*			
Yes	35(9.0)	33(94.3)	2(5.7)
No	353(91.0)	289(81.9)	64(18.1)

*History of chronic disease*			
Yes	85(21.9)	78(91.8)	7(8.2)
No	303(78.1)	244(80.5)	59(19.5)

*Family member working in healthcare*			
Yes	40(10.3)	34(85.0)	6(15.0)
No	348(89.7)	288(82.8)	60(17.2)

**Table 2:** Antibiotic resistance pattern of *S*. *aureus* isolated from patients attending University of Gondar Comprehensive Specialized Hospital, Ethiopia, 2018.

Drug name	Antibiotic resistance pattern (%)			
	*S. aureus (N* = 66)	MRSA (*N* = 14)	MSSA (*N* = 52)	iMLSB (*N* = 17)
Cefoxitin	14 (21.2)	14 (100)	—	3 (17.6)
Amoxicillin/clavulanate	15 (22.7)	8 (57.1)	7 (13.5)	6 (35.3)
Clindamycin	4 (6.1)	1 (7.1)	3 (5.8)	—
Erythromycin	26 (39.4)	8 (57.1)	18 (34.6)	17 (100)
Ciprofloxacin	4 (6.1)	3 (21.4)	1 (1.9)	1 (5.9)
Trimethoprim-sulphamethoxazole	29 (43.9)	12 (85.7)	17 (32.7)	6 (35.3)
Penicillin	63 (95.5)	14 (100)	49 (94.2)	16 (94.1)
Tetracycline	66 (100)	14 (100)	52 (100)	17 (100)
Nitrofurantoin	1 (1.5)	1 (7.1)	—	—
Chloramphenicol	—	—	—	—
Rifampin	6 (9.1)	3 (21.4)	3 (5.8)	1 (5.9)
Gentamicin	5 (7.6)	5 (35.7)	—	1 (5.9)
Doxycycline	57 (86.4)	13 (92.9)	44 (84.6)	15 (88.2)
Cloxacillin	10 (15.2)	6 (42.9)	4 (7.7)	3 (17.6)

**Table 3:** Bivariate and multivariate analysis of S. *aureus* isolated from patients attending University of Gondar Comprehensive Specialized Hospital, Ethiopia, 2018.

Variables	*Staphylococcus aureus*		Crude odds ratio (95% CI)	*p* value	Adjusted odds ratio (95% CI)	*p* value
	No (%)	Yes (%)				
*Sex*						
Male	52.2	47	1		1	
Female	47.8	53	1.23(0.725–2.094)	0.441	1.065(0.548–2.071)	0.852

*Age (years)*						
<1 year	33.2	19.7	1		1	
1–10 years	13.4	16.7	2.106(0.876–5.063)	0.096	1.265(0.457–3.497)	0.651
11–20 years	11.5	27.3	4.004(1.790–8.959)	0.001	1.762(0.394–7.882)	0.459
21–30 years	14	19.7	2.378(1.022–5.530)	0.044	2.642(0.265–26.382)	0.408
31–50 years	17.7	12	1.155(0.452–2.950)	0.763	1.184(0.099–14.106)	0.894
>50 years	10.2	4.5	0.748(0.201–2.786)	0.665	0.690(0.044–10.841)	0.792

*Marital status*						
Single	64	72.7	1		1	
Married	30.1	22.7	0.664(0.354–1.244)	0.201	0.937(0.234–3.757)	0.927
Divorced	2.2	3	1.226(0.247–6.089)	0.803	3.297(0.305–35.598)	0.326
Widow	3.7	1.5	0.358(0.045–2.817)	0.329	0.627(0.027–14.376)	0.770

*Family size*						
1–3	23.3	10.6	1		1	
4–6	60.6	71.2	2.582(1.118–5.967)	0.026	2.627(1.030–6.702)	0.043^*∗*^
>7	16.1	18.2	2.473(0.912–6.701)	0.075	3.892(1.169–12.959)	0.027^*∗*^

*Educational level*						
Illiterate	49.1	33.3	1.741(0.385–7.861)	0.471	1.104(0.093–13.181)	0.938
Primary	13.4	22.7	4.360(0.920–20.659)	0.064	2.886(0.292–28.558)	0.365
Secondary	18	24.3	3.448(0.737–16.132)	0.116	2.007(0.234–17.214)	0.525
College	11.6	16.7	3.618(0.739–17.725)	0.113	3.311(0.538–20.375)	0.197
University	7.8	3	1		1	

*Occupation*						
Government	14.9	13.6	1		1	
Merchant	4.7	6.1	1.422(0.383–5.286)	0.599	0.984(0.172–5.622)	0.985
Housewife	7.1	4.5	0.696(0.172–2.815)	0.611	2.414(0.319–18.288)	0.394
Labourer	6.8	6.1	0.970(0.269–3.492)	0.962	1.134(0.192–6.712)	0.889
Farmer	9.3	6.1	0.711(0.201–2.515)	0.597	1.386(0.162–11.886)	0.766
Unemployed	57.1	63.6	1.217(0.554–2.674)	0.624	2.071(0.192–22.324)	0.549

*Residence*						
Urban	58.4	72.7	1.901(1.059–3.413)	0.032	2.257(1.063–4.796)	0.034
Rural	41.6	27.3	1		1	

*Type of patient*						
Inpatient	73.9	80.3	1.439(0.747–2.772)	0.277	3.108(1.197–8.070)	0.020^*∗*^
Outpatient	26.1	19.7	1		1	

*Illness in the last 4 weeks?*						
Yes	46.3	66.7	2.322(1.331–4.052)	0.003	2.116(1.080–4.145)	0.029^*∗*^
No	53.7	33.3	1		1	

*Implanted Medical device*						
Yes	68.6	54.5	0.548(0.320–0.940)	0.029	0.605(0.302–1.213)	0.157
No	31.4	45.5	1		1	

*History of surgical procedure*						
Yes	6.8	9.1	1.364(0.530–3.506)	0.520	0.778(0.233–2.593)	0.683
No	93.2	90.9	1		1	

*History of hospital admission*						
Yes	10.2	3.0	0.274(0.064–1.170)	0.080	0.422(0.076–2.341)	0.324
No	89.8	97.0	1		1	

*History of chronic disease*						
Yes	24.2	10.6	0.371(0.163–0.846)	0.018	0.265(0.094–0.750)	0.012^*∗*^
No	75.8	89.4	1		1	

*Family member working in healthcare*						
Yes	10.6	9.1	0.847(0.340–2.107)	0.721	0.633(0.225–1.782)	0.386
No	89.4	90.9	1		1	

^*∗*^ *p* value < 0.05.

## Data Availability

The data supporting the conclusion of this article are available from the corresponding author upon reasonable request.

## Abbreviations

AICU: Adult intensive care unit
AST: Antimicrobial susceptibility testing
ATCC: American Type Culture Collection
CLSI: Clinical and Laboratory Standards Institute
cMLSB: Constitutive macrolide-lincosamide-streptogramin B
ERY: Erythromycin
FOX: Cefoxitin
iMLSB: Inducible macrolide-lincosamide-streptogramin B
MLSB: Macrolide-lincosamide-streptogramin B
MRSA: Methicillin-resistant staphylococcus aureus
MS Phenotype: Macrolides streptogramin phenotype
MSA: Mannitol salt agar
MSSA: Methicillin-sensitive staphylococcus aureus
S Phenotype: Erythromycin and clindamycin susceptible.
